# Meibomian gland dysfunction is highly prevalent among first-time visitors at a Norwegian dry eye specialist clinic

**DOI:** 10.1038/s41598-021-02738-6

**Published:** 2021-12-03

**Authors:** Reza A. Badian, Tor Paaske Utheim, Xiangjun Chen, Øygunn Aass Utheim, Sten Ræder, Ann Elisabeth Ystenæs, Bente Monica Aakre, Vibeke Sundling

**Affiliations:** 1 National Centre for Optics, Vision and Eye Care, Department of Optometry, Radiography and Lighting Design, Faculty of Health and Social Sciences, University of South-Eastern Norway, Notodden, Norway; 2 Department of Medical Biochemistry, Oslo University Hospital, Oslo, Norway; 3 Department of Ophthalmology, Oslo University Hospital, Oslo, Norway; 4 Department of Ophthalmology, Stavanger University Hospital, Stavanger, Norway; 5 The Norwegian Dry Eye Clinic, Oslo, Norway; 6 Department of Ophthalmology, Sørlandet Hospital Arendal, Arendal, Norway; 7 Department of Ophthalmology, Drammen Hospital, Drammen, Norway

**Keywords:** Medical research, Diseases, Eye diseases, Conjunctival diseases, Corneal diseases, Eyelid diseases, Lacrimal apparatus diseases

## Abstract

To investigate the prevalence of meibomian gland dysfunction (MGD) in patients presenting with subjective dry eye-related symptoms at their first-time consultation in a Norwegian specialized ocular surface clinic. Additionally, to explore the accuracy of the ocular surface disease index score (OSDI) as an extensively applied tool to assess the severity of dry eye symptoms and MGD diagnosis. Patients with subjective dry eye-related complaints (n = 900) attending the clinic for the first time, from 2012 to 2016, were included in the study. At the baseline, patients completed the OSDI questionnaire. Subsequently, objective clinical tests, including fluorescein break-up time (FBUT), Schirmer-I test, ocular surface staining (OSS), and meibomian gland function assessment using gland expressibility and meibum quality were performed. The association between MGD and its severity in relation to symptom severity defined by OSDI-score was examined. MGD was found in 93.8% of the study group. MGD prevalence was not significantly different between groups based on age (p = 0.302) or sex (p = 0.079). There was a significant association between severity of MGD and dry eye-related symptoms (p = 0.014). OSS was significantly higher in patients with severe symptoms (p = 0.031). Sensitivity and specificity of positive symptom-score (OSDI ≥ 13) for disclosing MGD were 85.5% and 30.4%, respectively. MGD was highly prevalent, not associated with age and sex. OSDI ≥ 13 had high sensitivity and high positive predictive value (PPV), but low specificity and negative predictive value (NPV) for disclosing MGD. This underscores the importance of meibomian gland assessment in patients with dry eye-related symptoms.

## Introduction

A well-functioning tear film is essential for the health and proper function of the ocular surface and to ensure ocular comfort and optimal vision^1,2^. Poor tear quality can result in ocular surface damage, which can negatively affect vision, quality of life^3,4^, as well as work productivity^5,6^. Dry eye disease^7^ is a multifactorial disease^7^, which may be aqueous-deficient and/or evaporative^8,9^. DED results in symptoms of ocular discomfort such as burning, foreign body sensation, pain, grittiness, itching, dryness, reduced or disturbed vision and instability of tear film^8,9^. Meibomian gland dysfunction (MGD) is defined as a chronic diffuse condition characterized by terminal duct obstruction, qualitative and quantitative changes in meibum, and symptoms including irritation, itching with subsequent lid rubbing, soreness, burning, and foreign body sensation^2,10–13^. MGD is the most common cause of evaporative dry eye and may be associated with aqueous-deficient dry eye^2,14,15^. MGD is believed to be one of the most common conditions encountered in ophthalmic practice^16^. Meibomian glands, located in the upper and lower eyelids^10^ produce meibum, the oily secretion that forms the outermost layer of the tear film that reduces the evaporation of the tear film and contributes to ocular surface lubrication^17–19^. Several studies have shown anatomical and functional alterations of the meibomian glands and lid margins accompanied by changes in quality and quantity of meibum^10,14,16,20–24^. Population-based studies have reported varying MGD prevalence worldwide, characterized by using different diagnostic definitions to diagnose MGD. In the US study of Salisbury, prevalence of dry eye was 3.5%, while among those with dry eye symptoms and signs 20.7% had MGD mentioned in the study as meibomititis^25^.

The prevalence of MGD is high in Asian populations^26–28^. In a Chinese study, the prevalence of subjective dry eye symptoms was 21%, however, using the presence of telangiectasia at the lid margin as definition of MGD resulted in a prevalence of 69%^26^. An Indonesian population-based study of subjects of Malay ethnicity found MGD prevalence to be 56.3%^27^. In a Japanese clinic-based, study MGD prevalence among patients scheduled for cataract surgery was 74.5%^28^. Reported MGD prevalence in European (21.9%)^29^ and U.S. (38.9%) populations is markedly lower^20^. Notably, the diagnostic criteria used to define and diagnose MGD across these studies varies considerably^16^. In this study, MGD diagnosis was made according to the report of the International Workshop on Meibomian Gland Dysfunction from 2011^11^.

The specialized ocular surface clinic in Oslo is unique in the Nordic region by being dedicated to diagnosis and treatment of patients with dry eye-related symptoms. To our knowledge, no studies have explored how prevalent MGD is in Norwegian patients with dry eye symptoms. The aim of this study was to explore how prevalent MGD is among the first-time visitors to the clinic and to explore the accuracy of symptoms in identifying MGD.

## Material and methods

### Study population, inclusion and exclusion criteria

This study had a cross-sectional design. Patients with dry eye-related symptoms at their baseline visit to the Norwegian specialized ocular surface clinic between January 2012 to January 2016, either self-referred, referred by optometrists, ophthalmologists, or general practitioners, were consecutively recruited to the study group. Patients who attended the clinic having followed their habitual dry eye treatment, either self-initiated or optometrists, ophthalmologists or general practitioners. Follow-up visitors or patients with a history of any ocular surgery in the past 12 months, conditions including ocular infections, or ocular allergy were not included.

### Prevalence

Prevalence is a frequency measure of morbidity that is defined as the proportion or rate of individuals who have a particular disease or condition at or during a particular time period. The frequency measure for the prevalence used here was proportion^30,31^. In this study, we defined the prevalence of MGD as the proportion of patients with MGD of the total number of patients in the study group, in the group as a whole or in age- and sex-stratified groups (Table 1) and in each symptom-category (Table 2).

**Table 1:** Distribution of the patients according to sex and age.

	Female	Male	Total
Age (years)	n_f_ (%)	n_m_ (%)	n_f+m_ (%)
≤ 20	9 (1.3)	4 (1.2)	13 (1.4)
21–39	123 (18.4)	84 (36.2)	207 (23.0)
40–59	252 (37.7)	73 (31.5)	325 (36.1)
60–79	259 (38.8)	65 (28.0)	324 (36.0)
≥ 80	25 (3.7)	6 (2.6)	31 (3.4)
Total	668 (100)	232 (100)	900 (100)

n, number of subjects in a stratum; f, female; m, male.

**Table 2:** Prevalence of MGD by sex and age groups.

Age strata (years)	Female	Male	Total
≤ 20	8/9 (88.9)	4/4 (100)	12/13 (92.3)
21–39	114/123 (92.7)	75/84 (89.3)	189/207 (91.3)
40–59	238/252 (94.4)	67/73 (91.8)	305/325 (93.8)
60–79	249/259 (96.1)	61/73 (83.6)	310/324 (95.8)
≥ 80	23/25 (92.0)	5/6 (83.3)	28/31 (90.3)
Total	632/668 (94.6)	212/232 (91.4)	844/900 (93.8)

N, total number of patients, females and males in age group; n, number of patients with MGD in sub-groups. (%) Prevalence value in percent for each subgroup.

### Clinical examinations, study parameters, and MGD diagnostic criteria

A comprehensive ophthalmic examination including assessment of subjective symptoms using Ocular Surface Disease Index (OSDI) questionnaire, and objective clinical signs/measurements including fluorescein tear break-up time (FBUT), ocular surface staining (OSS), Schirmer-I test (without anesthesia), and meibomian gland function evaluation were performed^32^. The OSDI is a 12-item self-reported symptom questionnaire that is one of the most used survey instruments to assess the severity of the subjective dry eye symptoms and its effects on visual function with a recall period of one week^33^. For assessing dry eye-related symptoms, we applied the OSDI-score, which ranges from 0 to 100, with four conventional symptom categories. The severity of subjective symptoms was categorized as; normal (0 ≥ OSDI-score < 13), in this study referred to as “normal symptom load”, mild (13–22), moderate (23–32), and severe (33–100) symptoms. The last three categories constituted the symptomatic group that is all patients with OSDI ≥ 13^34,35^.

Tear film stability was assessed by measuring FBUT after instillation of 5 µL of 2.0% fluorescein dye into the conjunctival sac with a micro-pipette, with values ≤ 10 s defined as unstable tear film^32,36^. The staining of interpalpebral cornea and conjunctiva with fluorescein constituted ocular surface staining (OSS) was used to evaluate ocular surface damage, which was graded according to the Oxford grading scheme, ranging on a scale from 0 to 15^32,37^. Tear production was assessed by performing the Schirmer-I test (without anesthesia).Wetting of the Schirmer strip after 5 min was measured, and values < 10 mm were defined as abnormal^32,36^.

Meibomian gland expressibility (ME) and meibum quality (MQ) were evaluated under a slit-lamp microscope by applying firm pressure using a cotton-tipped applicator onto the lower lid margin. ME was graded on a 4-point scale based on the number of expressible glands in the central five glands: grade 0, all five glands expressible; grade 1, 3–4 glands expressible; grade 2, 1–2 glands expressible; and grade 3, 0 glands expressible^11^. MQ was graded on a scale from 0 to 3 by examining the secretion of central eight meibomian glands in the lower lid: grade 0: clear meibum; grade 1: cloudy meibum; grade 2: cloudy with particles; and grade 3: inspissated or toothpaste-like meibum. A sum score for the central eight glands (range 0–24) was then calculated^11^. The severity of MGD was graded according to MQ and ME scores: grade 0, no MGD; grade 1, ME score = 1 and/or MQ ≥ 2–4; grade 2, ME score = 1 and/or MQ ≥ 4–8; grade 3, ME score = 2 and/or MQ ≥ 8 but < 13; and grade 4, ME score = 3 and/or MQ ≥ 13^38^. The criteria for MGD diagnosis in patients aged ≤ 20 years was score > 1 for either MQ or ME; and in patients aged > 20 years, a score of 1 for both MQ and ME or a score > 1 for either MQ or ME^11,38^.

OSDI-score as described above, was used as an instrument to assess the severity of dry eye-related symptoms^34,35^. Thus, we examined the accuracy of OSDI-score (≥ 13) by measuring its sensitivity and specificity in relation to disclose MGD that was the “disease” in focus for this study. It is worth underscoring that OSDI is not a tool to diagnose MGD rather to assess symptoms. OSDI measures dry eye-related symptoms. On this basis, the accuracy of OSDI measuring symptoms vis-à-vis presence or absence of MGD diagnosis was investigated, using binary classification of patients according to absence or presence of dry eye symptoms (normal symptom load OSDI < 13 and symptomatic OSDI ≥ 13) versus absence or presence of MGD. On this basis, using a contingency table reporting binary classification of patients according to the OSDI score (normal symptom load/OSDI < 13, and symptomatic category: OSDI ≥ 13) relative to absence or presence of MGD (Table 3). The sensitivity and specificity of OSDI score ≥ 13 in revealing MGD were determined. Subsequently, positive and negative predictive values (PPV, and NPV, respectively) were calculated based on the MGD prevalence^39^. PPV is defined as the probability of the subject/patient having MGD given that the OSDI score ≥ 13, that is having mild, moderate and severe dry eye symptoms. NVP is defined as the probability of the patient/subject not having MGD given that the OSDI score < 13, that is the patient having a normal symptom load. The formula and calculations of PPV and NPV are detailed in Eqs. (1) and (2), respectively.

1 $$\begin{aligned} & PPV = \frac{Senstivity \times Prevalance}{{Senstivity \times Prevalance + (1 - specificity) \times (1 - prevalence)}} = \frac{0.855 \times 0.938}{{0.855 \times 0.938 + (1 - 0.304) \times (1 - 0.938)}} = 0.9506 \\ & PPV = 95.06\% \\ \end{aligned}$$

2 $$\begin{aligned} & NPV = \frac{Specificity \times (1 - Prevalance)}{{(1 - Senstivity) \times Prevalance + Specificity \times (1 - prevalence)}} = \frac{0.3 \times (1 - 0.938)}{{(1 - 0.855) \times 0.938 + 0.304 \times (1 - 0.938)}} = 0.1487 \\ & NPV = 14.9\% \\ \end{aligned}$$

**Table 3:** Distribution of dry eye symptoms by sex and age, n (%).

OSDI groups	Sex			Age (years)				
	All (N = 900)	Female (n = 668)	Male (n = 232)	0–20 (n = 13)	21–39 (n = 207)	40–59 (n = 325)	60–79 (n = 324)	≥ 80 (n = 31)
Normal (OSDI < 13)	139 (15.4)	94 (14.1)	45 (15.4)	2 (15.4)	43 (20.8)	39 (12.0)	51 (15.7)	4 (12.9)
Mild (OSDI 13–22)	155 (17.2)	108 (16.2)	47 (20.3)	2 (15.4)	34 (16.4)	62 (19.1)	52 (16.0)	5 (16.1)
Moderate (OSDI 23–32)	141 (15.7)	99 (14.8)	42 (18.1)	3 (23.1)	27 (13.0)	51 (15.7)	54 (16.7)	6 (19.4)
Severe (OSDI ≥ 33)	465 (51.7)	367 (54.9)	98 (42.2)	6 (46.2)	103 (49.8)	173 (53.2)	167 (51.5)	16 (51.6)

OSDI, Ocular surface disease index; n, total number of patients in each subgroup; N, total number of patients in the study.

### Statistical analysis

Statistical analysis was performed using the Statistical Package for the Social Sciences (IBM SPSS Statistics, 24.0). The data are reported as frequencies, percentages with 95% confidence intervals (CIs), and means with standard deviation (SD). Pearson´s chi-square (χ^2^) tests were used for testing associations between categorical groups; MGD, MGD severity, sex, age, symptomatic and asymptomatic OSDI, and severity of dry eye symptoms. Correlations were tested using Spearman correlation. Kruskal–Wallis test was used to test for inter-group comparisons of clinical signs between different OSDI symptom categories. We used Dunn’s test with Bonferroni correction for post hoc analysis. A *p* value of < 0.05 was considered statistically significant.

### Ethical considerations

The study conformed to the Declaration of Helsinki, and informed consent was obtained from all patients. The Regional Committee for Medical & Health Research Ethics, Section C, South East Norway (REC) has reviewed the use of data material from the Norwegian Dry Eye Clinic. The REC found the research project "Evaluation of data from the Norwegian Dry Eye Clinic" to be outside the remit of the Act on Medical and Health Research (2008) and, therefore, can be implemented without its approval. A letter of exemption by REC is provided. Since 2013, extensive data have been collected and transformed into data sets to address specific research questions in various publications in The Norwegian Dry Eye Clinic.

## Results

### Demographics

**Figure 1:**
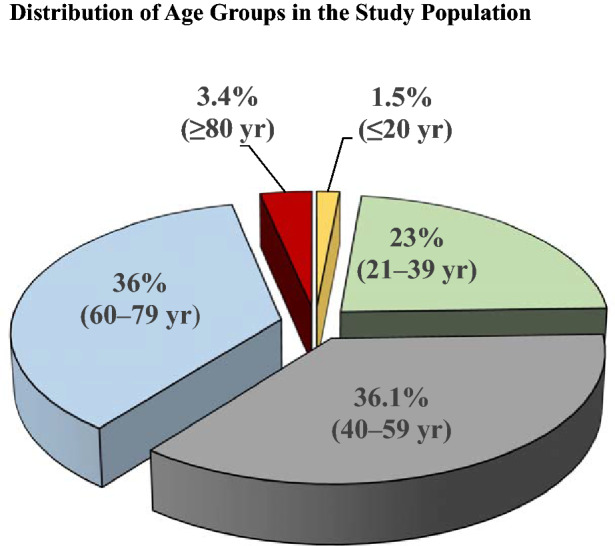
Age distribution of patients in the study population.

In total, 1027 patients were eligible for the study; complete data, including OSDI, ME, and MQ was available in 900 patients (87.6%) and were included in the analyses. The mean age of patients was 52 ± 16.7 years, 74.2% of patients (n = 668) were female. Figure 1 shows the age distribution in the study group and Table 1 presents the distribution of study subjects based on age and sex.

### Overall MGD prevalence and laterality of MGD

In total, 844 patients had MGD; giving an overall MGD prevalence of 93.8% (95% CI 90.0–95.3). Prevalence of MGD was not statistically significantly different between age groups and the sexes. MGD prevalence among female and male subjects was 94.6% (95% CI 92.9–96.3) and 91.4% (95% CI 97.7–95.0), respectively. Table 2 details the MGD prevalence data in the age- and sex-stratified groups. In 95.0% (95% CI 93.3–96.4) of the MGD patients the diagnosis was bilateral (unilateral: 5% (95% CI 3.6–6.7). With respect to sex 93.5% of female and 89.9% of male patients had bilateral MGD.

### OSDI score in relation to MGD diagnosis

In all, 84.6% (95% CI 82.2–87.0) of MGD patients had dry eye symptoms (OSDI score ≥ 13), and 15.4% (95% CI 13.1–17.8) had “normal symptom load” (OSDI score < 13). Prevalence of MGD in patients with dry eye symptoms (symptomatic group) versus patients with “normal symptom load” was significantly different (*p* = 0.001), 94.9% (95% CI (93.3–96.5), and 87.8% (95% CI 82.3–93.3%), respectively. Table 3 shows the sex and age-stratified OSDI-score distribution. In all, the percentage of patients with mild, moderate and severe dry eye symptoms in the group was 17.2% (95% CI 14.8–19.7), 15.7% (95% CI 13.3 – 18.1), and 51.7% (95% CI 48.4–54.9), respectively. MGD severity and dry eye symptom severity in the three subgroups (OSDI ≥ 13; mild, moderate, severe) were significantly associated (*p* = 0.014), higher MGD severity was associated with more severe dry eye symptoms. Table 4 presents MGD prevalence in groups stratified according to MGD severity and MGD prevalence for the four OSDI categories. The sensitivity and specificity of dry eye symptoms (OSDI-score ≥ 13) for disclosing MGD were 85.5% and 30.4%, respectively (Table 5). Positive predictive value (PPV) and negative predictive value (NPV) were determined to be 95.1% and 14.9%, respectively (Eqs. 1 & 2).

**Table 4:** Dry eye symptom severity and MGD Severity.

Dry eye severity grade	MGD severity grade				
	0 n = 56	1 n = 4	2 n = 167	3 n = 513	4 n = 160 (prevalence in the MGD, OSDI subgroups %)
1 Normal symptom load	17 (30.4)	1 (25.0)	22 (13.2)	70 (13.6)	29 (18.1)
2 Mild	9 (16.1)	1 (25.0)	38 (22.8)	87 (17.0)	20 (12.5)
3 Moderate	12 (21.4)	0 (0.0)	22 (13.2)	76 (14.8)	31 (19.4)
4 Severe	18 (32.1)	2 (50.0)	85 (50.9)	280 (54.6)	80 (50.0)

MGD, meibomian gland dysfunction; OSDI, Ocular Surface Disease Index. Grade 0: OSDI < 13, grade 1: OSDI = 13–22, grade 2: OSDI = 23–32, grade 3: OSDI =  ≥ 33–100, n: number, (%) percentage in each subgroup, n (%): number of subjects and prevalence in % in each subgroup.

**Table 5:** Contingency table for calculation of sensitivity and specificity of OSDI score in relation to MGD diagnosis.

Presence or absence of MGD diagnosis		OSDI test result		Total
		Normal symptom load OSDI-score < 13 (0–12)	Symptomatic OSDI-score ≥ 13 (13 – 100)	
MGD	No	17	39	56
MGD	Yes	122	722	844
Total	139	761	900	900

OSDI, Ocular Surface Disease Index; MGD, Meibomian gland dysfunction.

Sensitivity: 722/(122 + 722) = 722/844 = 0.855 or 85.5%.

Specificity: 17/(17 + 39) = 17/56 = 0.3035 or 30.4%.

### Relationship between OSDI-symptom severity categories and clinical signs; FBUT, OSS, and Schirmer

The mean values of FBUT, OSS, and Schirmer-I test for both eyes of all patients by the four OSDI categories are presented in Table 6. There was a statistically significant difference for OSS between the groups (p = 0.031). However, pairwise post hoc analysis using Dunn’s test with Bonferroni correction failed to identify statistically significant difference. The mean values for FBUT decreased with increasing OSDI scores; however, the intergroup difference did not reach statistical significance (p = 0.079). The Schirmer test was not significantly different between symptom groups (p = 0.138).

**Table 6:** Comparison of the average values for Schrimer-1 test, FBUT, and OSS of the two eyes of all study subjects and inter-group comparisons in relation to OSDI-based four symptom categories.

OSDI-based symptom category	N	Mean	SD	95% Confidence interval for mean		Kruskal–Wallis test (P value)
				Lower bound	Upper bound	
**Schirmer-I**						
Normal/normal symptom load (OSDI < 13: 0–12)	138	14.2	9.5	12.6	15.8	0.138
Mild (OSDI 13–22)	155	16.0	9.5	14.5	17.5	
Moderate (OSDI 23–32)	139	15.4	8.9	13.9	16.9	
Severe (OSDI 33–100)	462	14.5	9.3	13.7	15.4	
Total	894	14.9	9.3	14.3	15.5	
**FBUT**						
Normal/normal symptom load (OSDI < 13: 0–12)	139	5.6	4.3	4.8	6.3	0.079
Mild (OSDI 13–22)	155	5.0	3.6	4.5	5.6	
Moderate (OSDI 23–32)	141	5.1	4.4	4.4	5.9	
Severe (OSDI 33–100)	461	4.6	3.7	4.3	5.0	
Total	896	4.9	3.9	4.7	5.2	
**OSS**						
Normal/normal symptom load (OSDI < 13: 0–12)	139	1.5	1.7	1.2	1.8	0.031^❊^
Mild (OSDI 13–22)	155	1.4	1.7	1.2	1.7	
Moderate (OSDI 23–32)	141	1.4	1.7	1.1	1.7	
Severe (OSDI 33–100)	465	1.9	2.2	1.7	2.1	
Total	900	1.7	2.0	1.6	1.8	

FBUT, Fluorescein break-up time; OSS, Ocular surface staining; N, Number of individuals in each subgroup; OSDI, Ocular Surface Disease Index.

## Discussion

The vast majority of patients in this clinic-based study population had MGD. MGD was not associated with either age or sex. However, there was a significant association between the presence of MGD and subjective dry eye symptoms. Moreover, MGD severity and dry eye symptom severity also showed significant association.

Population-based studies of MGD prevalence have reported conflicting results with respect to age and sex. Whereas some studies have found MGD to be more prevalent in men^27,29^, others have not supported the association with sex^25^. Our results are in agreement with the latter study, as we did not find a relationship between MGD prevalence and sex. This result conflicts with findings in an Austrian dry eye clinic population, reporting MDG more frequently in females^40^.

The differences in prevalence can reflect population differences, and partly be because the MGD criteria used in different prevalence studies vary significantly. MGD prevalence studies, conducted as population-based studies, have shown substantial variation in MGD prevalence^20,25–27,29,41^. MGD is often clinically defined based on evaluation of meibomian gland obstruction, gland dropout, and abnormal gland secretions. In 26 studies reviewed by the International Workshop on MGD clinical trials subcommittee, 53.8% contained meibomian gland secretion assessment, 50% included the symptoms associated with DED, as entry, diagnostic or outcome criteria, and lid abnormalities such as telangiectasia was reported in 38.5%^42^.

The high prevalence of MGD in the present study likely reflects the sample population and diagnostic criteria. The study was conducted in a clinic-based population of first-time visitors with primarily subjective dry eye-related complaints and not in a general population; hence, a higher prevalence of MGD is expected. Nevertheless, the prevalence was higher than found in an Austrian dry eye clinic population^40^, a result that likely reflects variations in diagnostic criteria. In the present study, the diagnostic criteria for MGD were based on meibum expressibility and quality according to the international workshop on meibomian gland dysfunction: report of the diagnosis subcommittee^11,38^, whereas the Austrian study also included morphological changes as part of the diagnostic criteria.

Age is a risk factor of MGD^16^. In our study, age was not associated with MGD, possibly because the population were patients seeking help because of dry eye symptoms. However, the age distribution in our study was skewed, with the majority of patients with MGD (75%) being older than 40 years, corresponding with the age distribution found in the Austrian dry eye clinic study^40^.

In this research, MGD was significantly associated with the presence of dry eye symptoms (OSDI score ≥ 13) and symptom severity. MGD is the most common cause of evaporative dry eye disease^16,43,44^, resulting in dry eye-related symptoms^29,45–47^, and symptomatic MGD is characterized by symptoms of ocular discomfort such as irritation, soreness, redness of the eyes and eyelids, irritation, burning, itching of the eye, dryness, heavy or/puffy eyelids, and watery eyes^2,10,11,21,48–52^. The prevalence of MGD among first-time visitors to the Norwegian dry eye clinic reflects this pattern. However, the MGD prevalence was also high in the subgroup with normal symptom load. Of the total study subjects, 15.4% had a normal symptom load; nevertheless, from these patients’ perspectives, the symptoms were sufficiently severe to seek ophthalmological help. Consequently, high MGD prevalence even in the OSDI subgroup with normal symptom range (87.8%) indicates the substantial role of MGD in patients with dry eye-related symptoms of all symptom severities and multiple etiologies.

Some studies have indicated that asymptomatic MGD is more common than symptomatic MGD^28,29^ and most individuals with anatomical features of MGD are asymptomatic^25^. In Amano and colleagues' research conducted in a Japanese population, the overall MGD prevalence was 74.5%, whereas the prevalence of symptomatic vs asymptomatic MGD was 11.2% vs 63.3%^28^, respectively. These observations corresponds with studies that have indicated a large proportion of asymptomatic patients have some degree of MGD^28,29^. Patients with undetected MGD are at risk of gradual and continuous deterioration and progression to a chronic phase, with potentially irreversible changes in the anatomy and function of the meibomian glands. Consequently, deterioration of tear composition and quality, with aggravation of DED symptoms will follow^11,45,53–55^. Chronic and irreversible MGD is a major etiological factor of DED, which imposes substantial economic burdens on both patients and society^56^. The high percentage of patients with normal symptom load diagnosed with MGD in our study supports the premise that a substantial percentage of patients with MGD are asymptomatic, and may remain undetected, undiagnosed, and untreated. Dry eye-specific work-up should include assessment of meibomian gland function to detect MGD regardless of the symptoms, specifically to detect MGD in patients in the normal symptom load category.

DED is a multifactorial disorder of tears and the ocular surface^8^, and the two major etiological causes of dry eye disease are aqueous-deficient and evaporative dry eye, the former characterized by reduced tear volume measured by Schirmer-I test, and the latter by decreased tear film stability measured by FBUT^8,9^. The mean value of the Schirmer-I test in all OSDI groups was over the pathological cut-off value of 10 mm/5 min. However, a broad range of values was detected, indicating that aqueous deficiency was not the predominant objective clinical sign in our study population. Furthermore, FBUT, which indicates the stability of the tear film, was below the pathological cut-off of < 10 s in all OSDI groups. It is important to note that mild and moderate dry eye patients may show a broad range of FBUT values^57^. FBUT decreased with increasing OSDI severity, with the mean FBUT being lowest in the severe OSDI symptom group, although the intergroup difference did not reach statistical significance. Moreover, OSS was not significantly different between groups of patients with varying dry eye symptom severity. Considering that MGD is the most common cause of evaporative dry eye^2,14,15^, the very high MGD prevalence in the study supports the evaporative etiology as the more prominent cause of DED in the study population.

The present study highlights the poor diagnostics of patient self-reported symptoms. Dry eye symptoms (OSDI-score ≥ 13) correctly identified 85% of the patients with MGD. The predictive values of a test are dependent on the prevalence of the condition. The increased prevalence of MGD causes a high positive predictive value and a low negative predictive value. Our study shows that in a population with a high prevalence of MGD, dry eye symptoms correctly predict 95% of cases of MGD. However, lack of symptoms only correctly predicts 14% of cases without MGD. The low diagnostic accuracy of dry eye symptoms highlights the importance of meibomian gland assessment in the dry eye work-up.

## Conclusions

In sum, the overall MGD prevalence in this clinic-based Norwegian population was high and not associated with age and sex. Most patients had MGD with abnormal FBUT and normal Schirmer, supporting the significance of MGD as an underlying aetiological factor for evaporative dry eye disease. Our study shows that patient-reported dry eye symptoms have low diagnostic accuracy for MGD and that assessing the meibomian glands is essential and should be included as an integral part of the dry eye work-up to ensure correct diagnosis.
